# Where the children play: Gender gaps in recess physical activity by age and playground area

**DOI:** 10.1016/j.pmedr.2024.102699

**Published:** 2024-03-22

**Authors:** Matthew J. Barenie, Erin K. Howie, Kari A. Weber, Deboleena Thakur, Christopher M. Murphy, Michael R. Thomsen

**Affiliations:** a Center for the Study of Obesity, Fay W. Boozman College of Public Health, University of Arkansas for Medical Sciences, 4301 W. Markham, Slot 820, Little Rock, AR, USA; b Department of Health, Human Performance and Recreation, University of Arkansas, 155 Stadium Drive, Fayetteville, AR, USA; c Department of Epidemiology, Fay W. Boozman College of Public Health, University of Arkansas for Medical Sciences, 4301 W. Markham, Slot 82, Little Rock, AR, USA

**Keywords:** Physical activity, Pediatrics, Childhood obesity, Child health, Environmental strategies

## Abstract

**Objective:**

Recess contributes meaningfully to physical activity (PA), but recess time has declined. The study’s purpose was to report PA by age, gender, and playground feature to inform potential playground configurations more conducive to PA during recess.

**Methods:**

Using the System for Observing Play and Leisure Activity in the Youth, kindergarten through 5th grade recesses were observed on at least four days at four schools in Little Rock, Arkansas, United States during May 2023. Target playground features were ball courts, grass, pavement, play structures, and swings. To provide inferential analysis, a comparison of conditional means across playground features, age, and gender was conducted using linear regression with robust standard errors clustered by school.

**Results:**

3,356 playground scans (intercoder correlation = 0.93) were collected and aggregated by school, day, gender, age (grade), and target feature for a sample size of 292 observations. The gender gap was widest among 4-5th graders, 79 % (95 % CI: 72, 86) for males and 64 % (95 % CI: 58, 70) for females. Among females, PA was highest on swings [82 % (95 % CI: 77, 86)] and lowest on paved areas [56 % (95 % CI: 43, 69)]. Among males, it was highest on both swings [81 % (95 % CI: 75, 86)] and ball courts [83 % (95 % CI: 77, 89)] and lowest on grassy areas [64 % (95 % CI: 60, 67)].

**Conclusion:**

Swings, courts, and play structures were associated with a higher proportion of children being engaged in PA. Research is needed to identify whether physical improvements to facilitate access to these features increase PA.

## Highlights

• During recess 72% of observed children were engaged in physical activity.
• The gender gap (favoring males) was widest among older children (4th & 5th grades).
• For females, physical activity was highest on swings and lowest on paved areas.
• For males, physical activity was highest on swings and courts and lowest on grass.

## 1 Introduction

School is a vital setting to enhance student PA (Yu et al., 2021), and unstructured PA during school has been identified as an important way to improve overall PA and is one of five components within the Comprehensive School Physical Activity Program framework (Prevention, 2019). Recess contributes to children’s daily PA (Ridgers et al., 2006), which suggests a need to understand how playground design facilitates PA during recess. Understanding recess PA is important in light of evidence that the time schoolchildren have available for recess has been decreasing (Baines and Blatchford, 2019, Clevenger et al., 2019, Clevenger et al., 2023). More time in school is spent being sedentary despite the numerous and well-known benefits of PA (Clevenger et al., 2023, Weaver et al., 2016). Opportunities for school-based PA may be even lower for children in higher minority or lower socioeconomic schools due to fewer PA promoting policies or reduced resources for equipment and facilities (Stalsberg and Pedersen, 2010, Wolfe et al., 2020, Youth Online: High School YRBS - United States, 2005). Thus, strategies to improve PA in urban, lower socioeconomic schools are needed (Ariz et al., 2022 Oct, Monnat et al., 2017).

Research indicates between 12 and 50 % (Tyler et al., 2020, Ridgers et al., 2011, Gao et al., 2017) of recess time is spent in moderate-to-vigorous PA, and importantly, females (Anthamatten et al., 2014 Mar 1, Huberty et al., 2014 Feb 1) and older children (Escaron et al., 2021) have lower levels of PA both at recess and overall. Some research suggests gender gaps in PA are likely to differ by age (Escalante et al., 2011). The downward trend in PA levels may disproportionately affect older children due to increased emphasis on academic testing outcomes. Thus, there is a need to understand not only the factors that influence PA during recess, but also how PA differs by age and gender especially given diminishing recess time and more sedentary behavior of children at school (Baines and Blatchford, 2019, Clevenger et al., 2023).

Playground PA varies by the physical environment, including equipment provision, and activity type. While these factors have been identified as potential influences of children’s PA during recess, few studies have systematically observed PA across multiple ages and playground features. For instance, fixed equipment and markings have been investigated in children and adolescents, and the association with PA was inconclusive (Ridgers et al., 2012). In comparison, unfixed equipment (e.g., balls, skipping ropes) has been investigated only in children, with positive associations found (Ridgers et al., 2012). Systematic observation systems, such as the System for Observing Play and Leisure Activity in the Youth (SOPLAY) (Saint-Maurice et al., 2011, Poulos et al., 2022), have been used to understand the types of play and locations that occurs at recess. For example, a study observed higher levels of PA using SOPLAY on soft surface structured areas, but there were differences between genders in the effect of changing the surface type (Brink et al., 2010). Another study found both males and females to be more active on play structures compared to open fields (Farley et al., 2008). Finally, Raney et al. found differences in observed participation between males and females, with a higher percentage of males participating in traditional playground games and sports compared to females (Raney et al., 2019). Females participated more in gymnastics, dance, climbing and swinging (Raney et al., 2019). The activities and equipment used on the playground is likely to differ between genders due to socialized preferences for play (Blatchford et al., 2003). While these studies have found differences in play between males and females, none reported age-related differences, hence the importance of this work within a developmental context. Importantly, how PA participation varies by schoolyard location could help researchers understand the variable PA levels across gender and age, as well as the locations and structures that are most strongly associated with time spent in PA (Clevenger et al., 2019).

Prior work suggest climbing structures (Graham et al., 2021), open-play grassy areas (Frost et al., 2018) or areas conducive to organized sports (e.g., ball courts) (Massey et al., 2018) are most utilized for play. Because well-intentioned playground improvements are likely to alter playground features, there is a need to understand the role of these features and their impact on the intensity and duration of PA to ensure the improvements facilitate, not impede, PA opportunities. Thus, the goal is to report levels and differences in recess PA levels by age, gender, and target area in urban area schools. The socioeconomic status (SES) of the communities being served by these schools is low as evidenced by students eligible for free and reduced-price lunch comprised 93 % of all those enrolled. The data reported within provides a detailed description of baseline PA that will be incorporated into broader longitudinal study (Barenie et al., 2023) that will inform future playground improvements utilized during recess as well as how activity may differ by age and gender.

## 2 Methods

### 2.1 Setting

Data were collected during the Spring of 2023 for all children in kindergarten through 5th grade in four elementary schools within the Little Rock School District in Little Rock, Arkansas, United States. Two of the participating schools were selected due to their participation in a longer-term green schoolyard initiative that will involve playground improvements (Barenie et al., 2023). The other two schools were recruited based on their proximity and demographic similarities. The study received ethics approval from the University of Arkansas for Medical Sciences Institutional Review Board (#274741). The participating school district also reviewed and approved the study activities. Lastly, school-level administrators provided consent for this study protocol to occur at their respective schools. Because the data being collected were about target areas of the playground and not individual students, and the students were observed performing normal activities without researcher interaction, the approved protocol did not require guardian informed consent/assent for the SOPLAY observations herein.

### 2.2 Participants

Eligible children were those in kindergarten through 5th grade at each of the four schools. There were no additional exclusion criteria. All children present at their respective recess sessions were observed. School recess schedules varied between schools. Generally, 15–20-minute recesses were scheduled twice a day, once midmorning and again after lunch, for a minimum of 40 min of scheduled recess. Recesses included one or two grade levels on the playground simultaneously. School ground characteristics are outlined in Table 2. School A contains three play structures, two half-sized basketball courts, swings, and ample pavement space. School B’s layout consists of two play structures, two swing set locations, and one basketball court. School C contains three play structures throughout the schoolyard, along with a full basketball court, and a large grassy area. School D contains five play structures, swings, and two basketball courts.

**Table 1:** Intercoder agreement between observer pairs during recess observations in Little Rock, Arkansas, United States, May 2023.

Observer Pairs	Number of Scans	Intraclass Correlation Coefficient	95 % Confidence Interval
1	76	0.89	0.83–0.93
2	113	0.88	0.82–0.91
3	78	0.91	0.86–0.94
4	143	0.93	0.90–0.95
5	47	0.90	0.82–0.94
6	251	0.96	0.95–0.97
7	240	0.92	0.90–0.94
8	57	0.91	0.85–0.95
9	22	0.89	0.74–0.95
10	222	0.95	0.93–0.96
11	96	0.97	0.95–0.98
12	67	0.97	0.94–0.98
13	8	0.98	0.90–0.99
14	23	0.86	0.67–0.94
15	116	0.93	0.90–0.95
16	80	0.94	0.91–0.96
Note: A one-way average measures random effects model was used to calculate interclass correlation coefficients.

**Table 2:** Square meters (number of features) by target feature type and school in Little Rock, Arkansas, United States, May 2023.

**Feature Type**	**School**			
	**A**	**B**	**C**	**D**
Court	908 (2)	0	377 (1)	1,049 (2)
Grass	0	0	1,523 (1)	762 (1)
Pavement	866 (2)	267 (1)	0	197 (1)
Structure	755 (4)	552 (2)	704 (4)	1,064 (5)
Swings	121 (1)	220 (2)	0	258 (1)
Note: Google Earth Pro was used to estimate the areas in each playground feature type. School B did have courts and grass on the premises, but they were not used by children during the recess periods that were observed. Number in parentheses indicates number of individual target areas within each school’s playground.				

### 2.3 Observation method

The children were observed using the validated systematic observation tool, SOPLAY (Kinder et al., 2023), while in the playground environment during normal recess hours. Children at each school were observed on four different days. Each observation day included two schools observed simultaneously as required by the protocol for the broader multi-year study (Barenie et al., 2023). Prior to data collection, the research team mapped each schoolyard into target zones for SOPLAY measurement. The pre-selected target zones were defined as an area of the schoolyard designated for mostly a single dominant activity with natural borders, fencing, painted lines, and/or permanent structures. Following previous studies (Yu et al., 2021 Mar 1, Saint-Maurice et al., 2011, Raney et al., 2019 Apr 1, McKenzie et al., 2000 Jan, Zhang et al., 2020 Jun 1, Raney et al., 2023 Feb, Rahai et al., 2023 Mar); members of the study team visually scanned the pre-determined target zones of the playground and recorded the number of children engaged in various levels of PA at a given point in time. All pre-determined target zones were observed in the same order for each recess period as outlined in the seminal SOPLAY protocol (McKenzie et al., 2000). Before the scans, contextual characteristics of each target zone were coded, which included: accessibility, usability, presence of supervision, presence of school-provided equipment (e.g., balls, jump ropes) and whether play was formally organized under the instruction or facilitation of a school staff member. Each target area was scanned from left to right at an approximate time interval of one student per second. PA levels for each child were coded into three categories: sedentary, walking, or vigorous. After the PA counts, the most prominent activity taking place was recorded (e.g., swinging, tag/chasing, climbing). Independent scans were made by gender. Females were scanned first followed by males as described in the original SOPLAY protocol (McKenzie et al., 2000).

Eight trained individuals, including all the authors herein, conducted the recess observations. To ensure observer competency, observers were trained using standardized video samples and live-observation practice trials, however inter-observer reliability during training was not recorded due to differences in coder training schedules. Instead, a large number of reliability observations were used during the data collection. Specifically, we follow Poulos et al. who used a one-way average measures random effects model to assess inter-rater reliability for each coder pair (Poulos et al., 2022). SOPLAY observation began as soon as most children had access to the playgrounds and were engaged in the scheduled recess period. Observation continued until children were called to line up when recess ended. To measure intercoder correlation, most playground sessions were observed by two or more observers (less than 5 % of scans had a single observer). The observers used an R Shiny application (Shiny app) developed by the research team using the open-source R software environment to facilitate data collection (R Core Team, 2022.). The Shiny App allowed entry of basic climate data in addition to the SOPLAY data elements. The data-entry interface was a web browser on a mobile (iPadOS) device (iPad mini, 6th generation, Apple Inc, Cupertino, California, USA). Data were saved to a backpack webserver using a battery-powered travel router (Raspberry Pi 3B, Cambridge, UK). The webserver was a small laptop with a Linux operating system (Ubuntu 22.04) that had R Shiny Server software installed.

### 2.4 Statistical analysis

The number of children observed participating in walking and vigorous activity were summed for each scan. This sum was then divided by the total counts of children scanned to arrive at a percentage measure of overall PA for each scan similar to earlier work (Kinder et al., 2023). The SOPLAY scans were aggregated by school, date, gender, age, and target playground feature (see Table 3). Age was binned into three categories (lower elementary: (kindergarten & 1st grades), middle elementary (2nd & 3rd grades), and upper elementary (4th & 5th grades). After aggregation, the resulting analytic sample contained 292 data records on the percent of children engaged in PA across playground areas, gender, and age. Effects of gender, age group, and playground area on percentage of physically active children were estimated by linear regression with robust standard errors clustered by school. All statistical analyses were conducted using the R (v. 4.3.1) environment for statistical computing (Rahai et al., 2023). The standard for significance was set at an alpha level of 0.05.

**Table 3:** Descriptive Statistics for the Analytic Sample of Recess Observations at Four Elementary Schools in Little Rock, Arkansas, United States, May 2023.

Measure	N	Mean (SD) or %
**PA (proportion of children)**	292	0.723 (0.199)
**Gender**		
Female	149	51
Male	143	49
**Playground Area**		
Structure	92	32
Court	59	20
Grass	38	13
Pavement	59	20
Swings	44	15
**Grade Level**		
Lower (K-1)	88	30
Middle (2–3)	96	33
Upper (4-5)	108	37
**School**		
School A	76	26
School B	78	27
School C	60	21
School D	78	27
**Observation Day**		
16-May-2023	35	12
17-May-2023	47	16
18-May-2023	28	10
22-May-2023	41	14
23-May-2023	36	12
24-May-2023	24	8
30-May-2023	45	15
31-May-2023	36	12

Notes: Percentages may not sum to 100 due to rounding. The analytic sample is produced by aggregating over school, date, gender, grade level, and target feature.

## 3 Results

### 3.1 Sample

In total, 3,357 SOPLAY scans were conducted. Two observers were present for ∼72 % of recess periods, ∼24 % had three observers, and ∼4 % had a single observer. The overall scan-weighted average interclass correlation coefficient was *r* = 0.93. There were 16 unique pairs of observers totaling 1,639 scans across coder observation pairs used to assess reliability (Table 1). Based on climate recordings captured during the observations, the average temperature was 25.3 °C. The average humidity was 59.9 %. The average cloud cover was 48.3 % which indicated the presence of partial clouds, but no precipitation. Across all scans, accessibility, usability, supervision, formal organization, and presence of provided equipment was 98.7 %, 99.2 %, 99.1 %, 2.3 %, 29.8 %, respectively. Enrollment data from the Arkansas Department of Education indicated the total student enrollment for the four schools was 1,342. The enrollment-weighted average for student demographics across all schools was 86 % African American, 6 % white, 6 % Hispanic/Latino, and 2 % other race/ethnicity.

### 3.2 Recess observation data by area of playground and grade level

**Fig. 1:**
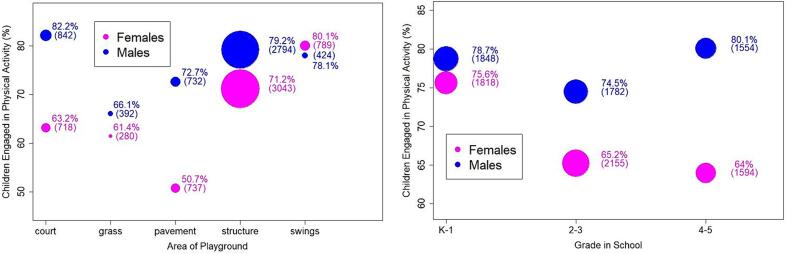
Descriptive presentation of recess observation data by area of playground (left) and grade in school (right). Size of the points is proportional to number of individuals observed in the scans. Point values are provided as percentages. Number of individuals is in parentheses.

Fig. 1 provides a descriptive overview of the observations by area of the playground and grade level. The highest numbers of children, both males and females, were observed on play structures (left panel of Fig. 1). Relative to other features, Fig. 1 shows high levels of PA on play structures relative to other areas of the playground. Exceptions were ball courts, where the proportion of children observed in PA was higher on average among males, and swings where the proportion of children observed in PA was highest among females. The right panel of Fig. 1 suggests a marked gender gap of ∼15 % PA between males and females in 4th and 5th grades. Slightly smaller numbers of upper-elementary children were observed during the observation periods.

Descriptive statistics for the analytic sample are reported in Table 3. Consistent with the data characterized above (Fig. 1), our analytic data set contains a larger number of observations on playground structures because all schools had structures. Table 3 also shows a larger number of records for upper elementary children in our analytic sample. This likely reflected that older children generally had more access to all areas of the playground resulting in more areas with non-zero counts being recorded during upper-elementary recess sessions. Overall, Table 3 indicates an average of 72 % of children observed were engaged in PA during recess.

### 3.3 Inferential analysis

Table 4 presents regression estimates used for inferential analysis. Among males, the percent involved in PA was significantly lower on grassy areas relative to play structures. Percentages across all other areas were not statistically different from play structures, the reference category. A greater percentage of females were observed in PA on swings relative to play structures (11.4 %, 95 % CI, 6.6 % to 16.2 %). There were no statistical differences among females across other areas relative to structures. Regression estimates by grade level (rightmost column of Table 4) indicates significantly fewer females are engaged in PA in upper elementary school grades (4th and 5th grades).

**Table 4:** Regression-estimated, Gender-specific Associations for Proportion of Children Involved in Physical Activity (PA) during Recess at Four Elementary Schools in Little Rock, Arkansas, United States, May 2023.

	Dependent Variable: PA	
	Model 1: Gender and Area	Model 2: Gender and Grade Level
Female (ref. Male)	−0.077	−0.028
	(−0.147, −0.007)	(−0.140, 0.084)
Male Grades 2 – 3 (ref. K − 1)		0.003
		(−0.089, 0.094)
Male Grades 4 – 5 (ref. K − 1)		0.034
		(−0.057, 0.125)
Female Grades 2 – 3 (ref. K − 1)		−0.036
		(−0.081, 0.009)
Female Grades 4 – 5 (ref. K − 1)		−0.088
		(−0.145, −0.031)
Male Court (ref. Structures)	0.047	
	(−0.030, 0.124)	
Male Grass (ref. Structures)	−0.142	
	(−0.168, −0.116)	
Male Pavement (ref. Structures)	−0.044	
	(−0.129, 0.041)	
Male Swings (ref. Structures)	0.025	
	(−0.042, 0.092)	
Female Court (ref. Structures)	−0.019	
	(−0.131, 0.094)	
Female Grass (ref. Structures)	−0.061	
	(−0.131, 0.008)	
Female Pavement (ref. Structures)	−0.146	
	(−0.307, 0.014)	
Female Swings (ref. Structures)	0.114	
	(0.066, 0.162)	
Observations	292	292
F Statistic	6.349 (df = 9; 282)	4.048 (df = 5; 286)

Note: 95% confidence intervals, in parentheses, are based on robust standard errors clustered by school.

**Fig. 2:**
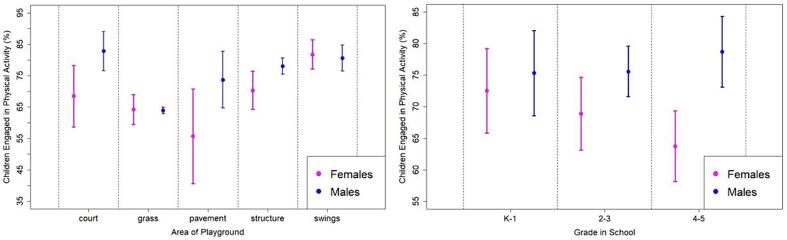
Conditional mean PA proportions with 95 percent confidence intervals imputed from linear regression analysis. The model at left involved a regression of PA on grade and area with grade by area interactions. The model at right involved a regression of PA on gender and grade with gender by grade interactions. In each model, standard errors were clustered by school.

To facilitate visualization of differences across areas and grade levels, conditional mean PA levels based on these regression models with accompanying 95 percent confidence intervals (CI) are presented in Fig. 2. The proportion of PA among males remains stable throughout all grade levels. Among females, there is near parity with males in lower elementary school followed by a decline leading to statistically significant gender gap by the time children are in upper-elementary grades. Among children in 4th and 5th grades 15 % fewer females were observed in PA relative to males (-15 %, 95 % CI, −25.2 % to −4.8 %).

## 4 Discussion

This work’s goal was to assess recess PA among children across grades and gender. An additional goal is to provide data to inform the design and remodeling of playgrounds that may increase intensity and duration of PA during recess to promote PA as part of the overall Comprehensive School Physical Activity Program framework. We observed the proportion of children observed in PA on swings (∼81 % PA), ball courts (i.e., basketball court) (∼76 % PA) and play structures (∼74 % PA) as the three most physically active playground areas. Other similar literature corroborates our findings on courts (Spengler et al., 2011) and swings (Amholt et al., 2022 Oct 2, Sargisson and McLean, 2013) as features favorable to PA during recess.

The present data adds to the robust literature (Ariz et al., 2022 Oct, Escaron et al., 2021, Ridgers et al., 2012 Sep, Saint-Maurice et al., 2011, Graham et al., 2021, Lemberg et al., 2023 Apr, Pawlowski et al., 2015 Aug 1, Tanaka et al., 2019, Woods et al., 2015) that females tend to spend less time in PA during recess than males. Speculatively, differences in social activities as children age result in males being more likely to socialize through sport and thus getting more PA (Blatchford et al., 2003). The observation females become less active as they age (Ariz et al., 2022) is supported herein. The proportion of females in 4th and 5th grades was significantly lower than those in kindergarten and 1st grades. Other studies agree (Ridgers et al., 2011, Lopes et al., 2006) suggesting females in older grades prioritize socialization at recess versus active play (Marouf et al., 2016), or an absence of age-appropriate playground equipment may result in less PA among older female children (Jerebine et al., 2022). Additional research indicates a disparity between the PA children want to do at recess versus what they are allowed to do (Jerebine et al., 2022), this may partially explain the recess observation data reported herein. Not measured directly in our study, study team members anecdotally reported supervising staff restricting certain playground activities. To address this PA age gap from worsening, involvement of children in decisions about playground rules and interventions may be considered.

The largest number of children were observed on play structures, which the schoolchildren used for climbing, sliding, tag games and other active behavior. Consistent with earlier findings, play structures, were highly popular areas on the observed school grounds (Raney et al., 2023 Feb, Stanton-Chapman et al., 2018) and have been associated with higher levels of PA (Saint-Maurice et al., 2011, Graham et al., 2021, Escalante et al., 2014 Apr 1). One factor that may explain the popularity of play structures is their large footprint at each and every school. Furthermore, all the schools had multiple climbing structures to use, while the availability of other features varied across schools. Moreover, when supervising staff were shorthanded, children were commonly confined to areas comprised of these structures.

Courts, specifically basketball courts, were also present at all four schools and proportionately had the highest level of observed PA for males at 83 %. Observed activities included basketball, cheer/dance, football, and other ball games. Equipment was provided on nearly 30 % of occasions. On one occasion when equipment was not provided, children improvised equipment as observers noted the use of a sandal for a football game. Loose equipment and sports equipment has been shown to be associated with an increase in PA levels (Roberts et al., 2013). Our observations that there was a higher proportion of PA among males on courts aligns with previous studies because males have been observed to engage in more competition (Blatchford et al., 2003) and to prefer sport activities (Woods et al., 2012). While specific equipment wasn’t objectively measured, a majority of equipment seemed to be sports based (i.e., basketballs and footballs), conducive to activities preferred by males (Lemberg et al., 2023) and may explain some of our findings on the gender gap. However, other data show no significant differences in gender-specific preferences in recess activities, aside from sports (Lemberg et al., 2023). For example, a playground predominately consisting of soccer and basketball courts may not be the most conducive playground setting for PA among females. Swings, based on our data and others (Raney et al., 2019) may be a better playground renovation for females as well as renovations that provide ample room and play structures for activities like tag and climbing (Lemberg et al., 2023).

The playground area with the highest proportion of observed PA for females was swings. Three of the four schools contained swings. Swings have been shown to be preferred (Lemberg et al., 2023 Apr, Caymaz et al., 2018, Springer et al., 2013 May), and study team members repeatedly overheard children expressing a desire for additional swings and more opportunities to use this playground area. Thus, future playground design may consider including and/or expanding swing structures to increase PA in females.

The area observed with the lowest proportion of children engaged in PA was paved areas (absence of permanent structures, e.g., basketball hoop) (∼65 %). Our finding was similar to Andersen and colleagues (Andersen et al., 2015). In our study, paved areas included 4-square, hop-scotch, and areas of open asphalt. Speculatively, the lack of PA in these areas could have been due to the lack of equipment provided, lack of clearly painted lines/shapes/zones for play (playground markings) or the development appropriateness for all ages. Motor skill development varies widely among elementary school aged children, with younger grades developing fundamental motor skills and less ability to integrate basic motor skills into more complex games which may explain the influence of motor skills on PA (Logan et al., 2015). Earlier studies have found incorporating new playground markings on pavement increased PA (Baquet et al., 2018), but this is not a universal finding (Raney et al., 2023 Feb, Wong et al., 2023 Apr 3). Some studies observed equipment availability did promote moderate and vigorous PA (Roberts et al., 2013 May 1, Verstraete et al., 2006 Aug 1). However, a systematic review highlighted the insufficient evidence playground markings and game equipment to increase the PA of schoolchildren (Escalante et al., 2014), which suggest incorporation of swings and play structures should be prioritized over pavement areas.

Another explanation for lower proportion of PA in pavement areas may be due to recess observations taking place in mostly warm and sunny conditions making these areas uncomfortable as asphalt is a strong heat collector (Aletba et al., 2021). The study could not determine the extent to which temperature reduced use of paved areas due to limited variations in temperature across data collection days. However, the research team did repeatedly overhear sedentary children complain about hot weather being reason for not playing games. Observers also noted children aggregated in areas with shade trees and, if trees were not available, under pavilion-like structures. Anecdotal observations of heat adversely affecting play herein and evidenced elsewhere (Ridgers et al., 2018 Jul, Ridgers et al., 2010 Oct 12) suggests future research on the influence of shade structures (i.e., shade sails, trees) on PA is needed. One study showed shaded areas increased PA (Lanza et al., 2022), while another study did not (Poulos et al., 2022).

The more grassy, natural target areas observed had a smaller proportion of children engaged in PA (∼64 % PA) compared to courts, structures, and swings in contrast to Dyment and colleagues which observed a high proportion of students engaged in PA in green areas (Dyment et al., 2009). Speculatively, two schools had no grass accessible during recess and one of the remaining schools had a large water drain in the main grassy area that was not conducive to play and became muddy after storms. The dominant activity of the schoolyard grassy area was soccer and football-based games. However, males were sometimes observed in a football-based game with brief bursts of vigorous activity accompanied by standing behaviors other times, which may have led to lower observed PA given the SOPLAY observation method. Other activity was sedentary socialization under trees, possibly to escape the heat. Grassy areas remained neutral on our PA findings for females and relative to play structures was negative for males, whereas other studies indicated more varied and intense activity (Lemberg et al., 2023 Apr, Bikomeye et al., 2021 Jan, Bohnert et al., 2022 Jun). Other groups found natural areas in schoolyards did not attract children (Zhang et al., 2021) and tended to be less inviting for more intense play (Van Dyck et al., 2022). Furthermore, Brussoni and collaborators found a decrease in moderate-vigorous PA when natural materials were added to the schoolyard (Brussoni et al., 2017). Conversely, addition of grassy areas in the schoolyard may assist thermal comfort (Shashua-Bar et al., 2011), potentially reduce injury versus playing on pavement surfaces, and provide an environment for PA via sports and other games.

The schools within are of low SES and findings have shown higher SES schools provide more equipment but were also less likely to have a playground than lower SES schools (Van Dyke et al., 2018). Quality of space and access to PA opportunities vary across SES status as some studies find an association between SES and PA (Wolfe et al., 2020), while other data do not (Ridgers et al., 2012). To our knowledge, the precise nature and strength of these relationships have not yet been studied, hence SES and PA is currently inconclusive.

Our findings illustrate preferences and patterns of schoolchildren’s PA during recess. For this study, well-trained observers reliably collected observation data on PA (indicated by strong intercoder correlation), for an understudied population of children using SOPLAY, an extensively used recess observation tool (Poulos et al., 2022, Kinder et al., 2023). However, this study had limitations. Our data collection took place in a single month and all four locations were in the same municipality. This prevented comparison of results between different temperatures and other geographical locations. While we present both the total number of students observed in each target area and the average intensity instead of a single combined total PA variable, this is like most work with SOPLAY (Kinder et al., 2023), which combines walking and vigorous categories. Furthermore, the current work is part of a larger, community study (Barenie et al., 2023) working with education and government stakeholders, to improve playground space, and a simpler metric (proportion of children engaged in PA) is likely more interpretable for the broader audience.

## 5 Conclusions

There is a need to prioritize playground renovations that may facilitate greater intensity of play among females, especially older elementary females. Playground improvements that facilitate access to swings, courts, and play structures may increase opportunities for PA and the percentage of active schoolchildren during recess. Future research should focus on clarifying which specific playground features and areas are most effective in increasing PA, especially older and female schoolchildren.

## 6 Ethics approval and consent to participate

Ethical approval for this study has been received by the University of Arkansas for Medical Sciences Institutional Review Board (Protocol #274741). All methods were carried out in accordance with relevant guidelines and regulations.

## Funding

This research was supported by the National Institute on Minority Health and Health Disparities of the National Institutes of Health under award number R01MD018192. The content is solely the responsibility of the authors and does not necessarily represent the official views of the National Institutes of Health. Funding sources had no role in the writing of this manuscript or the decision to submit it for publication.

## CRediT authorship contribution statement

**Matthew J. Barenie:** Writing – original draft, Visualization, Supervision, Project administration, Investigation, Formal analysis, Conceptualization. **Erin K. Howie:** Writing – review & editing, Supervision, Resources, Methodology, Conceptualization. **Kari A. Weber:** Writing – review & editing, Supervision, Methodology, Investigation, Conceptualization. **Deboleena Thakur:** Writing – review & editing, Investigation. **Christopher M. Murphy:** Writing – review & editing, Investigation. **Michael R. Thomsen:** Writing – review & editing, Supervision, Software, Resources, Project administration, Methodology, Investigation, Funding acquisition, Formal analysis, Data curation, Conceptualization.

## Declaration of competing interest

The authors declare that they have no known competing financial interests or personal relationships that could have appeared to influence the work reported in this paper.

## Data Availability

The authors intend to release study data through an open repository at the end of the four-year study.
